# Rainfall seasonality and pest pressure as determinants of tropical tree species' distributions

**DOI:** 10.1002/ece3.383

**Published:** 2012-09-27

**Authors:** Jennifer L Baltzer, Stuart J Davies

**Affiliations:** 1 Department of Biology, Wilfrid Laurier University75 University Ave. W, Waterloo, Ontario, N2L 3C5, Canada; 2 Center for Tropical Forest Science – Arnold Arboretum Asia Program, Harvard UniversityBoston, Massachusetts, 02131; 3 Department of Botany, MRC-166, Smithsonian InstitutionP.O. Box 37012, Washington, District of Columbia, 20013-7012

**Keywords:** Drought, global change, herbivory, Kangar-Pattani Line, species distributions, tropical forests

## Abstract

Drought and pests are primary abiotic and biotic factors proposed as selective filters acting on species distributions along rainfall gradients in tropical forests and may contribute importantly to species distributional limits, performance, and diversity gradients. Recent research demonstrates linkages between species distributions along rainfall gradients and physiological drought tolerance; corresponding experimental examinations of the contribution of pest pressure to distributional limits and potential interactions between drought and herbivory are limited. This study aims to quantitate differential performance and herbivory as a function of species range limits across a climatic and floristic transition in Southeast Asia. Khao Chong Botanical Garden, Thailand and Pasoh Forest Reserve, Malaysia straddle the Kangar-Pattani Line. A reciprocal transplantation across a seasonality gradient was established using two groups of species (“widespread” taxa whose distributions include seasonally dry forests and “aseasonal” taxa whose distributions are limited to aseasonal forests). Growth, biomass allocation, survival, and herbivory were monitored for 19 months. Systematic differences in performance were a function of species distribution in relation to rainfall seasonality. In aseasonal Pasoh, aseasonal species had both greater growth and survivorship than widespread species. These differences were not a function of differential herbivory as widespread and aseasonal species experienced similar damage in the aseasonal forest. In seasonally dry Khao Chong, widespread species showed higher survivorship than aseasonal species, but these differences were only apparent during drought. We link this differential performance to physiological mechanisms as well as differential tolerance of biotic pressure during drought stress. Systematic decreases in seedling survival in aseasonal taxa during drought corresponded with previously documented physiological differences and may be exacerbated by herbivore damage. These results have important implications for tropical diversity and community composition in light of predicted increases in the frequency and severity of drought in hyperdiverse tropical forests.

## Introduction

Boundaries of geographic ranges are thought to be constrained by biotic pressures (e.g., competition) at low latitudes where abiotic conditions are favorable, whereas higher latitude range limits are thought to be imposed by severe abiotic conditions (e.g., drought) (Dobzhansky 1950; MacArthur 1972; Sax 2001). Within tropical forests, the seasonality of precipitation increases with distance from the equator with corresponding changes in species composition and reductions in richness (Clinebell et al. 1995). Drought and pests have been proposed as the major abiotic and biotic factors, respectively, determining tree species' distributional limits in the lowland tropics and, as such, may contribute importantly to tropical forest dynamics and diversity gradients.

Tree species distribution patterns vary substantially with water availability in tropical forests (Borchert 1998; Veenendaal and Swaine 1998; Pyke et al. 2001; Toledo et al. 2012). Periods of low rainfall, during which evapotranspiration exceeds precipitation and soil water available to plants is reduced, is the main climatic constraint in tropical lowland forests (reviewed in Borchert 1998). Experimental evidence indicates that drought sensitivity is an important mechanism underlying these distributions (Tyree et al. 2003; Baltzer et al. 2005, 2008; Engelbrecht et al. 2007). However, physiological and anatomical adaptations for tolerating low soil water potentials have also been demonstrated to trade off against performance (e.g., safety vs. efficiency tradeoffs sensu Hacke et al. 2006), suggesting that under conditions of ample water availability, drought-tolerant species will be at a competitive disadvantage.

Interactions between plants and pests also contribute to species distributions (Fine et al. 2004) and diversity maintenance (Wright 2002). Tropical wet forests are hypothesized to suffer greater herbivore pressure than drier forests (Connell 1971; Coley and Barone 1996; but see Dirzo and Boege 2008). Such a gradient may thus contribute to species distributional patterns along rainfall gradients within the tropics, although little evidence exists to directly test this hypothesis (Coley and Barone 1996; Brenes-Arguedas et al. 2009). If drier sites experience less herbivore pressure, then species from those sites should have lower investment in defense (Coley and Barone 1996) and consequently experience greater damage and reduced performance when grown in wetter forests characterized by higher rates of damage.

**Figure 1:**
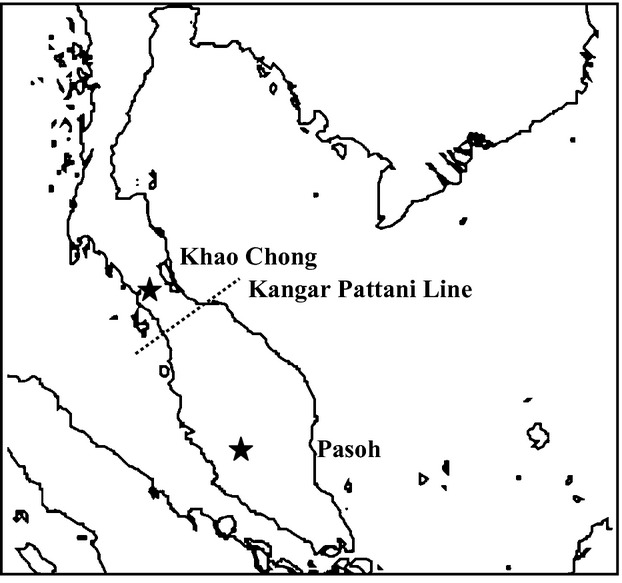
Map of the location of the Kangar-Pattani Line (dashed line) and locations of the reciprocal transplant gardens: Pasoh Forest Reserve (Pasoh; 2°58′N, 102°18′E) and Khao Chong Peninsular Botanical Garden (Khao Chong; 7°34′N, 99°47′E), denoted by stars (
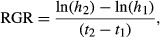
). Forests to the north of the KPL experience seasonal rainfall while to the south are considered aseasonal. Modified from Baltzer et al. (2008).

The geographic range limits of many species are expected to shift in response to climate change; in the wet tropics, one of the major predicted changes is increasing frequency and severity of drought (Malhi and Phillips 2004; Williams et al. 2007). However, such predicted changes in precipitation will not occur in isolation; to facilitate predictions of species responses to such changes, we have to understand the effects of and interactions among important biotic and abiotic determinants of distribution in tropical tree species. Predicting potential range shifts based on forecast climatic changes alone (i.e., climate envelope models) may not suffice as biotic drivers can substantially impact species' responses to predicted environmental changes (Clark et al. 2011). Here, we examine the role of drought and herbivory in the distribution of trees in relation to the shift from an aseasonal to a seasonally dry climate across one of the world's most dramatic floristic transitions: the Kangar-Pattani Line (van Steenis 1950) (see Methods; Fig. 1).

We employed reciprocal transplantation at seasonally dry (Khao Chong) and aseasonal (Pasoh) locations along the climatic transition (Fig. 1). In addition to impacting plant performance due to resource limitation, drought can alter plant palatability and defense and the resulting performance of insect herbivores (Mattson and Haack 1987); thus, precipitation gradients are potentially confounded with gradients of pest pressure (Coley and Barone 1996) necessitating such an approach. Further to this, recent work along this gradient has been limited to common garden quantitation of inherent differences in physiological and morphological traits contributing to drought tolerance. Plants may show substantial plasticity in response to drought irrespective of inherent trait differences and may employ various strategies (e.g., drought avoidance via changes in biomass allocation; reviewed in Ludlow 1989) for surviving drought periods that do not involve the physiological tolerance mechanisms previously demonstrated (Baltzer et al. 2008, 2009). Understanding the mechanistic basis of this floristic transition is essential to our understanding of the potential responses of this hyperdiverse region to predicted climate change-associated shifts in the frequency and/or severity of moisture deficits. Seedlings of 16 tree species having either distributions restricted to the aseasonal forests (aseasonal species) or distributions that traverse the seasonality gradient (widespread species) were planted together at each location, and growth, biomass allocation, survivorship, and herbivory quantitated. We predicted that: (1) widespread species would outperform aseasonal species in seasonally dry Khao Chong due to differential drought tolerance, but that this pattern would be reversed in aseasonal Pasoh due to abovementioned tradeoffs between drought tolerance and competitive ability; and (2) a gradient of herbivory would be evident with greater damage in aseasonal Pasoh and widespread taxa would be more susceptible to damage, which should result in reduced performance of widespread species in aseasonal Pasoh (pest pressure gradient hypothesis). Our predictions assume that drought will be the main factor determining northern limits of aseasonal species and that biotic pressures (competitive differences, differential herbivore resistance, or both) will contribute to performance differences in wetter, aseasonal forests where these species co-occur.

## Methods

### Study sites

The Kangar-Pattani Line (KPL) (van Steenis 1950) bisects the Thai-Malay Peninsula near the political border and corresponds with a major floristic (turnover of approximately 575 genera; Van Steenis 1950) and climatic transition (from aseasonal rainfall to a 2–3-month seasonal drought with little change in annual precipitation). We conducted this study in the Pasoh Forest Reserve (2°58′N, 102°18′E; Fig. 1) and Khao Chong Peninsular Botanical Garden (7°34′N, 99°47′E). Pasoh is located approximately 400 km south of the KPL and is classified as aseasonal, evergreen forest, whereas Khao Chong is located approximately 100 km north of the KPL and is classified as seasonally dry, evergreen forest (Fig. 1). Species richness in Pasoh's Center for Tropical Forest Science (CTFS) 50-ha forest dynamics plot is roughly 490 species/ha for stems >1-cm diameter at breast height. In the CTFS 24-ha plot in Khao Chong, tree species richness is approximately 300 species/ha. Climatic differences at the two sites are largely related to precipitation with Khao Chong experiencing a 2–3-month seasonal drought from January to March (here, drought is defined as any month receiving <100 mm of rainfall). In Pasoh, minimum/maximum temperatures and mean annual precipitation are 22.7/33.2°C and 1950 mm, respectively, whereas in Khao Chong, these are 22.7/32.6°C and 2700 mm, respectively. Although Khao Chong has higher total annual rainfall than Pasoh, it is the annual rainfall minima and its length that affects the physiognomy of tropical forests rather than the annual amount (Borchert 1998). Hereafter, Pasoh will be referred to as the aseasonal forest and Khao Chong as the seasonally dry forest for clarity. The study period covers two seasonal droughts at Khao Chong. The 2006 drought was relatively weak with ample rainfall in the months preceding the dry season (365, 874, 88.1, and 112.1 mm in November – February 2006, respectively), whereas in 2007, the months preceding the dry season had low rainfall and February 2007 received next to no rainfall (109, 119, 103, 3.2 mm for November – February 2007, respectively).

### Plant material and distributions

We collected seeds of 16 tree species (Table S1) from July to September 2005 in both forests. Seeds of two or more individuals were obtained for each species with the exception of *Parashorea densiflora* for which only one fruiting tree was located (Table S1). As a goal of our sampling scheme was to facilitate comparisons of congeneric species pairs differing in geographic distribution, the numbers of adults available as seed sources were sometimes low (e.g., if the only available fruiting congener had one or few fruiting adults).

We classified each species as widespread, aseasonal, or seasonal based on its distribution with respect to the KPL. As we were only able to find seed for two “Seasonal” species at the time of collection, we have grouped the data into two distributional categories: “Widespread” refers to species whose distributions include the seasonally dry forests north of the KPL; and “Aseasonal” refers to species whose distributions are restricted to aseasonal forests south of the KPL. The ranges were based on plant taxonomic records. Primary sources were the *Tree Flora of Malaya* (Whitmore 1972), *Flora Malesiana* (van Steenis 1950), and *Flora of Thailand* (Smitinand and Larsen 1970), all of which list the states/provinces and other countries in which the species occurs. Additional range data were collected from other reliable sources (e.g., Symington 2004; van Welzen and Chayamarit 2005).

Seeds were germinated and seedlings grown in polybags (15.2 cm diameter × 22.9 cm height) at each location filled with local forest soil. Seedlings were grown in shade structures covered in neutral density shade cloth providing approximately 15% full sunlight for 2–4 months prior to out-planting. Seedlings were hand-watered every evening if diurnal rainfall had not been sufficient. Prior to out-planting into the forest plots, 10 seedlings of each species were randomly selected for biomass harvest. For each harvested seedling, polybags were cut away and excess soil was gently removed by hand. Shoots were removed at the root collar, leaves were separated from stems and each part placed in a labeled paper envelope. Root systems were gently hand-washed in a bucket of water to remove soil following which the wash water was sieved to avoid loss of roots during this process. The clean root system was placed in a labeled paper envelope. All samples were weighed following oven drying at 70°C for 3–4 days.

### Experimental design

Ten sites within each forest were selected for experimental plots. Approximately, 75-m^2^ plots were established in small, naturally occurring gaps, avoiding slopes. We used small gaps rather than understory locations to avoid confounding impacts of potential differences in light availability between forests. Seasonally dry forests tend to have higher understory light availability due to shorter canopy stature and lower basal area (Malhi et al. 2002; Losos 2004), and seasonal drought can impact the degree of deciduousness in the canopy resulting in greater seasonal variation in light transmission. Within each plot, 4–7 replicates of each species were planted in a randomized block design with 0.50-m spacing and 1-m strips separated planting blocks. In total, the experiment consisted of 1780 seedlings. Encroaching canopy branches were removed to expand the canopy opening and minimize light variation among plots and between forests. Ground vegetation was hand-cut at ground level and removed from plots.

Planting was conducted during November 2005, giving seedlings in Khao Chong approximately 2 months in the ground before the onset of the dry season. During planting, polybags were cut off and seedlings planted maintaining the soil in which they had germinated and grown in order to minimize damage to roots during planting. Uniquely labeled tags were attached loosely around the root collar. Plots were revisited 2 weeks after planting and dead individuals were replaced. The final harvest was conducted in June 2007; therefore, the experiment was in the ground for 19 months including two dry seasons in Khao Chong.

### Survivorship

Survival censuses were conducted seven times over the course of the experiment. Censuses started in December 2005 and recensuses were conducted in February, June, September, and November 2006, and February and June 2007. Only seedlings that survived at least 2 weeks post-transplant were included in analyses.

### Growth and biomass allocation

During each census, height along the main stem of every live seedling was measured. During the last census, all live seedlings were harvested. A large block of soil was cut around each seedling and gently lifted out by hand, thus avoiding loss of roots. Upon seedling excavation, excess soil was gently removed by hand, the root system was cut at the root collar, and shoots and roots were separately sealed in labeled plastic bags for transport to the lab where the harvest protocol described above was used to quantitate leaf, stem, and root dry masses for each seedling. Height and biomass relative growth rates (RGR_h_ and RGR_b_, respectively) were calculated as follows:


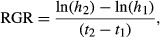


where, RGR is relative growth rate, h_1_ and h_2_ are height (or mass for RGR_b_) at time 1 and 2, respectively, and t_2_−t_1_ is the time between measurements. Leaf, stem, and root mass ratios were calculated as the mass of the organ of interest divided by total plant mass, and root:shoot was calculated as root mass divided by shoot mass.

### Herbivory

To examine differences in herbivory between forests and as a function of species distributional differences, we quantitated herbivory attributable to chewing insects four times over the course of the experiment. Herbivory censuses were conducted at approximately 6-month intervals (December 2005, May and October 2006, and June 2007). As we were measuring attached, living leaves, we used clear, gridded sheets to consistently estimate percent damage per leaf. During each census, every expanded leaf on every plant was measured. Missing leaves were excluded from damage censuses, as the cause of leaf loss could not be determined and natural leaf senescence should not be considered damage; thus, our estimate of herbivory is somewhat conservative.

### Statistical analyses

Relative growth rates, biomass allocation, survival, and %herbivory over the entire study period months) were analyzed using mixed effects models (nmle package; Pinheiro and Bates 2004). The design included two fixed effects: forest type (two levels: seasonal or aseasonal) and species distribution (two levels: widespread or aseasonal); and two random factors: species (16 levels) and plot (20 levels, 10 plots per site). Herbivory data were log_10_-transformed and seedling survival data were square root-arcsin-transformed to meet model assumptions. In all cases, a significant forest type × distribution interaction was detected. Significant interactions may mask the ability to describe main effects; therefore, data were re-coded creating a single, combined variable with four levels. For our questions, this was preferable to splitting the data as we are interested both in differences between distributional groupings within a forest as well as comparison between the two forest types. All analyses were also run excluding the two “seasonal” species from the widespread category; this had no effect on the results (data not shown).

To examine determinants of differential seedling survival and changes in survival through the experimental period, we employed the logistic regression models (glm function with a binomial distribution and logit link). The response variable was survivorship (0 = death, 1 = survival) and the predictor variables included size metrics (plant height and number of leaves in the previous census), species distribution (two levels: widespread and aseasonal), %herbivory in the previous census, and the interaction between herbivory and distribution to assess whether there were differential impacts of herbivory on species having differing distributions with respect to rainfall. Forests and census intervals were analyzed separately to allow for an examination of the contribution of rainfall seasonality to potential differences in survival between distributional groupings. For each forest, we ran three separate logistic regression models rather than a single survivorship model with rainfall as a covariate because time to event (death) was not unique from rainfall in the previous census; thus, in order to examine the impact of rainfall seasonality on distributional differences in survival, censuses had to be analyzed separately.

To account for phylogeny on all abovementioned traits, we conducted randomized block Analysis of Variance (ANOVAs) using congeneric species pairs having differing distributions in relation to the KPL. There were five such pairs in our dataset (Table S1). In these analyses, forest, distribution, and their interaction were fixed variables and genus was the random variable. Dependent variables included RGR, seedling survival, and %herbivory. Where a significant interaction existed, we split the data by forest type to allow for the examination of the main effect of distribution on the variable in question. Herbivory data were log_10_-transformed and seedling survival data were square root-arcsin-transformed to meet model assumptions. All analyses were conducted using R (v. 2.11.1; The R Foundation for Statistical Computing, Vienna, Austria).

## Results

### Forest and distribution-based performance differences

**Figure 2:**
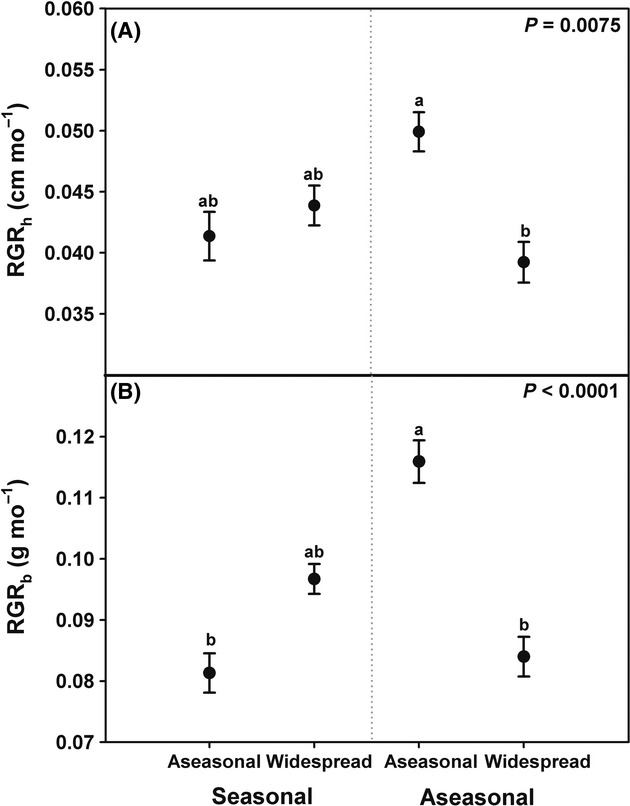
Tree seedling growth rates on a height (RGR_h_, A) and biomass (RGR_b_, B) basis. as a function of species distribution and forest type. Differences in seedling growth rates were analyzed using linear mixed models with the fixed factor being a single combined category for forest and distribution; random factors included species and plot. Lower case letters indicate results from a Tukey test (*P* < 0.05). Aseasonal species have distributions restricted to aseasonal forests (Pasoh), whereas widespread species have distributions including seasonally dry forests (Khao Chong). Values represent averages across species ±1 SE over 19 months. Note that the axes do not start at zero.

Both height and biomass growth rates were comparable between the two forests (Fig. 2; Table 1). When grown together in the seasonally dry forest, widespread and aseasonal species performed similarly (Fig. 2). In the aseasonal forest, however, aseasonal species had significantly greater relative growth rates on both a height and biomass basis in comparison with widespread species (Fig. 2). Aseasonal species also showed significantly greater biomass, but not height growth when grown in the aseasonal forest while widespread species did not differ in either trait when comparing forests.

**Table 1:** Summary of linear mixed models for height- and biomass-based relative growth rates, root, stem, and leaf mass ratios, root-shoot ratio, %survival, and %herbivory over the entire study period (19 months). The design included two fixed effects: forest type (two levels: seasonal or aseasonal) and species distribution (two levels: widespread or aseasonal); and two random factors: species (16 levels) and plot (20 levels, 10 plots per site). Herbivory and root–shoot ratio data were log_10_-transformed and seedling survival data were square root-arcsin-transformed to meet model assumptions. Because of significant forest × distribution interaction terms in all analyses, data were re-coded creating a single, combined variable (ForDist) with four levels. Significant *P*-values are bolded

Trait	DF_num_	DF_den_	*F*-value	*P*-value
Height growth	3	1048	4.01	**0.0075**
Biomass growth	3	1071	5.93	**<0.0001**
Root mass ratio	3	1071	5.18	**0.0015**
Leaf mass ratio	3	1071	8.82	**<0.0001**
Stem mass ratio	3	1071	1.69	0.167
Root–shoot ratio	3	1071	3.77	**0.0104**
Survivorship	3	1060	6.32	**<0.0001**
Herbivory	3	1060	5.84	**<0.0001**

**Figure 3:**
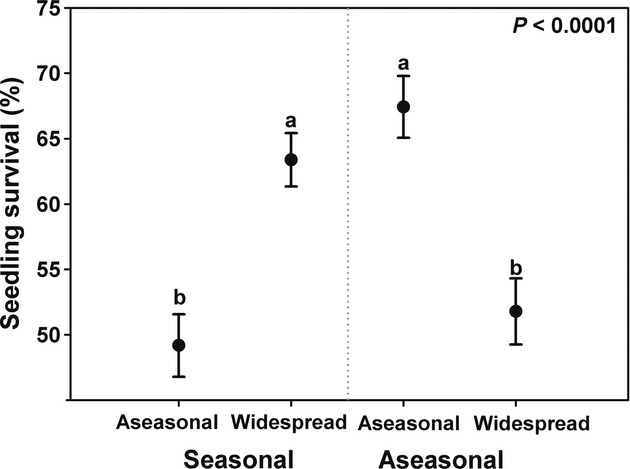
Tree seedling survival as a function of species distribution and forest type. Analytical description and definition of distributional categories follow Fig. 2.

Forest type impacted survivorship in both widespread and aseasonal species. Widespread species had lower survivorship over the course of the experiment in the aseasonal forest when compared with the seasonally dry forest (Fig. 3). The reverse held true for aseasonal species. Likewise, patterns of survivorship as a function of species distributions were reversed between the two forests (Fig. 3). In the aseasonal forest, survivorship of widespread species at the end of the experiment was about 20% lower than that of seedlings of aseasonal species. Widespread species growing in the seasonally dry forest had significantly higher survivorship than aseasonal species (Fig. 3).

These distribution-based differences in performance held even when phylogeny was accounted for through comparisons of congeneric species pairs (Figs. S1, S2). In the seasonally dry forest, species distribution was not a significant predictor of height growth, whereas in the aseasonal forest, aseasonal species grew significantly faster than their widespread congeners (Fig. S1). Although genera varied substantially in survivorship when grown in both forests, systematic differences existed, such that widespread species had higher survivorship in the seasonally dry forest and lower survivorship in the aseasonal, compared with their aseasonal congeners (Fig. S2).

### Herbivory

**Figure 4:**
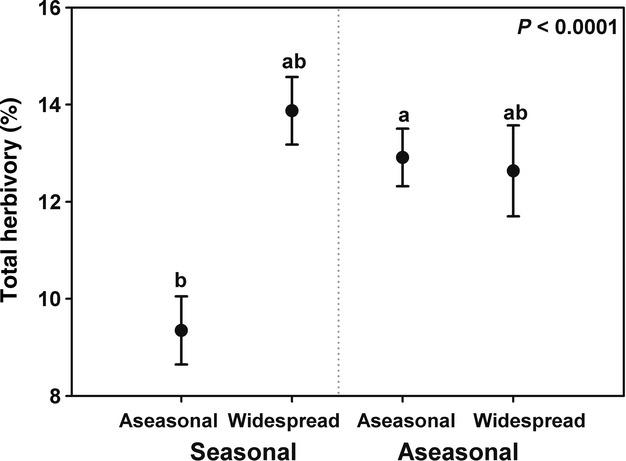
Herbivory as a function of species distribution and forest type. Analytical description and definition of distributional categories follow Fig. 2.

In general, herbivory was fairly minimal over the course of the experiment (Fig. 4). Herbivory rates were less than 2% per month (data not shown) and total standing herbivory at the end of the experiment was generally less than 15%, with the exception of a couple of species (Fig. S3). Aseasonal species growing in the aseasonal forest had significantly greater standing herbivory at the end of the experiment than when they were grown in the seasonally dry forest (Fig. 3). However, the same pattern did not exist for the widespread species. These patterns held in the corresponding congeneric analysis. In the aseasonal forest, congeneric pairs showed similar total herbivory, whereas in the seasonally dry forest, widespread species incurred significantly greater herbivory than their aseasonal congeners (Fig. S3).

### Biomass allocation

**Figure 5:**
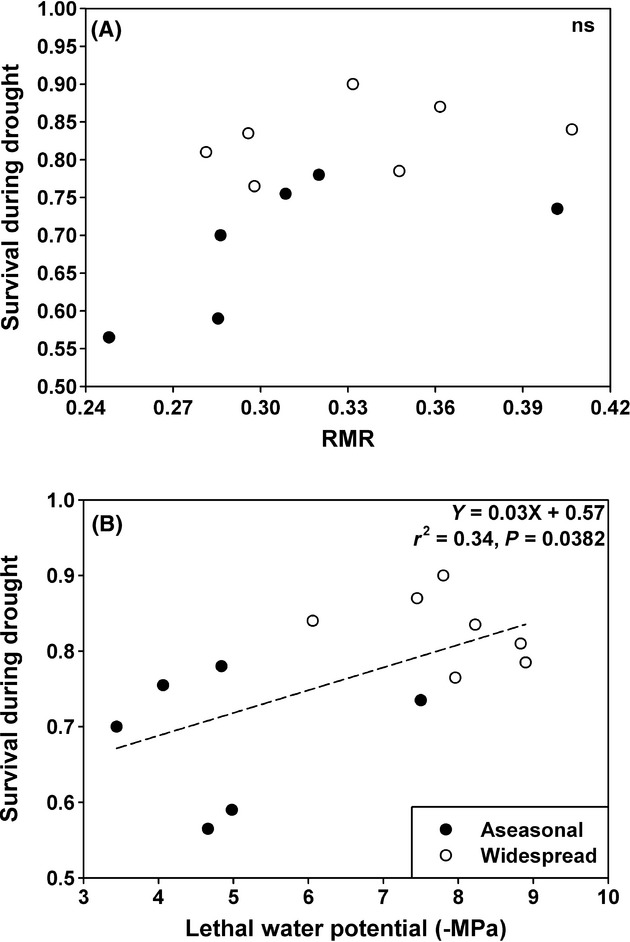
Relationship between survival during drought and (A) root mass ratio (RMR) or (B) lethal water potential (i.e., tissue water potential prior to death; Baltzer et al. 2008). Values represent species' mean values. Statistics correspond with ordinary least squares regression analysis. Symbols are coded by distribution type, but regression lines were fit using the combined dataset. Definition of species distributions follows Fig. 2.

In terms of biomass allocation, there was a tendency for widespread species to allocate preferentially to root biomass (Figs. 5A, S4), although the only statistically significant difference supporting this pattern existed in terms of RMR in seedlings growing in the aseasonal forest (Fig. S4). The one consistently significant pattern in the allocation data was that aseasonal species showed significant biomass allocation shifts between the two forests for all measured variables; aseasonal species had lower RMR and higher LMR when growing in the aseasonal forest (Fig. S4).

### Predictors of survivorship

We were interested in characterizing the relative contributions of distribution, herbivory, and their interaction to observed differences in survivorship at each forest site and whether this differed across census intervals. In the seasonally dry forest, the first and last censuses (A and C) included seasonal drought, thus census corresponds with drought in the seasonally dry forest. Our logistic regression models showed that one or both size metric (height and number of leaves) was positively related to survival in both forests and in each census interval (Table 2). In the aseasonal forest, species distribution was a significant predictor of survival across all censuses with aseasonal species having higher survivorship than widespread species (Table 2). In the third census, herbivory was also a significant negative predictor of survival, but there was no interaction between distribution and herbivory (Table 2). In the seasonally dry forest, distribution was a strong significant predictor of survival in censuses A and C with widespread species having higher survival than aseasonal species. In the censuses where it was a significant predictor (censuses including drought, A and C), distribution was a stronger predictor of survival in the seasonally dry forest compared with the aseasonal forest. In the third census, herbivory was a significant predictor of survival and there was a significant interaction between distribution and herbivory (Table 2). To determine the direction of this interaction, we split the dataset by distribution for that interval and ran the same logistic model without the distribution or interaction terms. In this post-hoc analysis of herbivore impacts in the seasonally dry forest, herbivory was a significant predictor of survival for the aseasonal species (*P* < 0.0001), but not for the widespread species (*P* = 0.0555).

**Table 2:** Logistic regression model of individual seedling survival as a function of seedling height, number of leaves, and herbivory in the previous census and distribution (Dist) and its interaction with herbivory (Dist × Herb). Parameter estimates (standard error) and *P*-values are given with significant predictors of survivorship bolded. Aseasonal (Pasoh) and seasonally dry (Khao Chong) forests were analyzed separately. To examine the contribution of rainfall seasonality to survival, separate models were run for each census period; in the seasonally dry site, census intervals A and C included the seasonal drought (January – April)

		Census A		Census B		Census C	
				
Forest	Coef.	Estimate	*P*	Estimate	*P*	Estimate	*P*
Wet	Height	−0.01 (0.02)	0.5153	0.01 (0.02)	0.5723	0.02 (0.02)	0.1392
	Leaves	0.09 (0.03)	**0.0043**	0.23 (0.05)	**<0.0001**	0.06 (0.03)	**0.0460**
	Dist	−0.42 (0.17)	**0.0117**	−0.33 (0.13)	**0.0390**	−0.50 (0.28)	**0.0732**
	Herbivory	−0.08 (0.05)	0.1324	−0.02 (0.02)	0.4249	−0.06 (0.02)	**0.0007**
	Dist × Herb	0.04 (0.06)	0.4867	0.01 (0.03)	0.8465	0.02 (0.02)	0.2760
Seasonal	Height	0.07 (0.02)	**<0.0001**	0.08 (0.03)	**0.0099**	−0.01 (0.01)	0.5113
	Leaves	0.15 (0.05)	**0.0012**	0.59 (0.09)	**<0.0001**	0.08 (0.04)	**0.0303**
	Dist	0.60 (0.15)	**<0.0001**	−0.22 (0.33)	0.5032	0.70 (0.30)	**<0.0001**
	Herbivory	−0.05 (0.04)	0.1572	−0.02 (0.02)	0.2909	−0.11 (0.02)	**<0.0001**
	Dist × Herb	0.06 (0.05)	0.1792	−0.01 (0.03)	0.6912	0.08 (0.03)	**0.0047**

## Discussion

Our data provide experimental evidence supporting previously demonstrated correlative links between species distributions and rainfall in tropical forests (Condit 1998; Oliveira and Fontes 2000; Pyke et al. 2001). Strong, systematic patterns of differential performance were evident between aseasonal and widespread species. Specifically, aseasonal species showed enhanced performance in terms of both growth and survivorship when growing in aseasonal Pasoh compared with widespread species. This is in keeping with earlier, plot-based analyses from Pasoh in which species distribution with respect to the KPL was used as a predictor of performance in co-occurring adult trees; widespread species had significantly lower growth rates in Pasoh compared with aseasonal species (Baltzer et al. 2007). Such patterns support the hypothesized relationship between broad geographic ranges and greater tolerance of abiotic stress (Morin and Chuine 2006), leading to reduced competitive ability in the widespread species or “climatic generalists”. Previous work with the same suite of species provides evidence of systematic, trait-based differences between the two groups with widespread species characterized by a conservative suite of functional traits conducive to stress tolerance, but trading off against productivity (Baltzer et al. 2008, 2009). In seasonally dry Khao Chong, growth-based differences disappeared suggesting that differential growth is not a major factor contributing to species' northern distributional limits in this region. In contrast, widespread species showed higher survivorship in the seasonally dry forest compared with aseasonal species. Interestingly, there was evidence that the negative response of aseasonal species to drought in seasonally dry Khao Chong was exacerbated with increasing herbivore damage. Our data suggest that both drought and herbivory are contributing to distribution-based survivorship differences; this interaction between biotic and abiotic stresses will have important implications for forest community composition and dynamics in the face of predicted climatic changes including increased frequency and severity of drought.

### Drought as a determinant of tree species distributions

Our survivorship analysis suggests that the seasonal drought in Khao Chong is driving distribution-based differences in survivorship (Table 2; Fig. S5). In census intervals that included seasonal drought, aseasonal species had much lower survival compared with widespread species (Table 2; Fig. S5). Brenes-Arguedas et al. (2009) showed corresponding patterns of differential droughtbased mortality in wet versus dry forest species when grown together in the dry forests on the Pacific side of the Isthmus of Panama. They demonstrated a peak in mortality of the wet distribution species, following the drought in the dry forest and using supplemental watering identified drought-induced differences in soil water availability as the cause of distribution-based differences in mortality (Brenes-Arguedas et al. 2009).

A number of traits can contribute to differential responses of species to drought via delay, avoidance, or tolerance (reviewed in Ludlow 1989; Breda et al. 2006). Inherent differences and/or plasticity in patterns of biomass allocation can both increase rooting volume and decrease evapotranspirative surface area, thereby delaying the onset of drought (Ludlow 1989). In this study, differences in biomass allocation were minimal; no systematic differences existed between widespread and aseasonal taxa that could explain systematic differences in survivorship (Fig. S4), which is further demonstrated by the lack of a significant relationship between root mass ratio (RMR) and survivorship during drought (orship during drought (Fig. 5A). Differences in drought deciduousness can also factor strongly into differential survivorship in response to water stress as drought deciduous species simply shut down during drought, thus avoiding its potentially detrimental effects (reviewed in Ludlow 1989). In this study, this is not contributing to differences in performance; none of the study species employ drought deciduousness at the seedling/sapling stage (J. L. Baltzer, pers. obs.). At the larger forest scale, drought deciduousness is similarly not likely to be contributing strongly; both forests are evergreen and neither has a considerable drought deciduous component.

Previous results from the same suite of species demonstrate strong, systematic differences in physiological desiccation tolerance and corresponding traits (Baltzer et al. 2008, 2009). Species whose distributions include the seasonally dry forests employ a strategy conferring abiotic stress tolerance including wood anatomical traits aimed at hydraulic safety, lower physiological rates enhancing resource conservation, and traits conferring foliar physiological desiccation tolerance; in combination, these conservative traits contribute to enhanced desiccation tolerance in widespread species (Baltzer et al. 2008, 2009). These systematic differences correspond with differential drought-related patterns of survivorship and thus likely contribute importantly to differential distributions (or the maintenance of such differences) of species with respect to drought in this region. For example, we had previously quantified lethal water potential (minimum water potential associated with living tissue) in the same species (Baltzer et al. 2008), and there exists a positive relationship between survival during seasonal drought in Khao Chong and lethal water potential across species (Fig. 5B). Similar linkages have been demonstrated between field performance, desiccation tolerance, and species distributional limits along the strong precipitation gradient on the Isthmus of Panama (Engelbrecht et al. 2007) suggesting a more general importance of physiological desiccation tolerance in shaping tropical tree species communities along precipitation gradients.

### The contribution of herbivory to species distributions

Theory predicts that pest pressure should be greater in wetter forests compared with seasonally dry forests, and that species from wetter forests should thus invest more heavily in defensive traits (Connell 1971; Coley and Barone 1996). Under this hypothesis, by extension, we could make the prediction that “specialized” aseasonal species restricted to the wetter, aseasonal forests of Malaysia should be better defended against the postulated greater pest pressure in aseasonal forests, then the more generalist, widespread species. Our data did not support these contentions. First, herbivory rates and total herbivory were fairly low in both forests, regardless of distributional grouping (Figs. 4, S3). No difference in total herbivory or herbivory rate existed as a function of forest type (Fig. 4); in other words, we detected no gradient in pest pressure. Furthermore, distributional grouping did not systematically predict the degree of foliar damage in our study (Fig. 4), even when phylogeny was accounted for (Fig. S3). Had a sufficient sample of “Seasonal” species (i.e., those with southern distributional limits to the north of the KPL) been available, this conclusion might have been different. It is possible that co-occurring widespread and aseasonal species invest comparably in foliar defense, and as foliar defensive compounds were not quantified, we cannot be certain one way or the other. However, a comparable reciprocal transplant study along the precipitation gradient in Panama similarly found no evidence for distribution-based differences in susceptibility to herbivores, and concluded that herbivores do not exclude dry distribution species from wetter forests at the seedling stage (Brenes-Arguedas et al. 2009). The correspondence of these two experimental studies puts into question the generality of this proposed mechanism of species filtering within the tropics. There is also the possibility that these forests 500 km apart differ substantially in their herbivore communities and that local herbivores may avoid taxa to which they are unaccustomed. Taxonomic records suggest, however, that Malaysia and Peninsular Thailand show strong similarities in the herbivore communities (e.g., <5% of Lepidoptera found in Malaysia, but not Peninsular Thailand; D. Lohman, pers. comm.). Large plant genera provide a continuously distributed resource over hundreds of kilometers, which when combined with the fact that many herbivores are able to use multiple species (i.e., are generalist) leads to low turnover of tropical herbivore communities over large areas (Novotny et al. 2007). Thus, there is a low likelihood that systematic distribution-based differences in the use of our study species by herbivores biased our results or masked underlying gradients.

The one systematic difference that we did find in terms of herbivory was reduced damage in aseasonal species in the seasonally dry forest (Figs. 4, S3). Aseasonal species showed lower total herbivory in the seasonally dry forest than in the aseasonal forest across species (Fig. 4). Between congeneric pairs, aseasonal species showed significantly lower total herbivory in the seasonally dry forest than their widespread congeners (Fig. S3). A likely explanation of this difference given the lack of differentiation in herbivory between widespread and aseasonal species in the aseasonal forest is that greater water-stress symptoms in aseasonal species are resulting in systematic reductions in palatability (reviewed in Huberty and Denno 2004) and a resulting avoidance of aseasonal species by herbivorous insects in the seasonally dry forest. Although plant nitrogen is often increased under conditions of water stress via increases in N-rich osmoregulatory compounds, which was previously thought to increase the attractiveness of water-stressed plants to herbivores (Plant Stress Hypothesis; White 1969), concentrations of allelochemicals also often increase and turgor pressure and tissue water content both decrease. A recent quantitative review of herbivore response to water-stressed plants demonstrated that all insect-feeding guilds do in fact respond negatively to water-stressed plants despite increases in tissue nitrogen (Huberty and Denno 2004). Stress-induced changes in water content, tissue toughness, and/or allelochemistry affect an insect's access to and utilization of nitrogen, thus reducing attractiveness in comparison with less stressed plants (Scriber 1977; McMillin and Wagner 1995; Inbar et al. 2001; Huberty and Denno 2004). Previous evidence supports the contention that widespread species will be less stressed in response to drought than aseasonal species (Baltzer et al. 2008); thus, observed distribution-based differences in herbivory may be attributable to water-stress driven differences in palatability between the two groups, a difference that disappears when grown together in aseasonal Pasoh.

Despite a lack of support for the pest pressure gradient hypothesis, we provide evidence that herbivory may be contributing to species distributional differences in this region, but that the observed mechanism differs from that predicted by theory. In both forests, herbivore damage was a significant negative predictor of seedling survival though only in the third census period, which in the seasonally dry forest corresponded to a strong seasonal drought. While no distribution-based differences existed in the aseasonal forest (i.e., no significant interaction term; Table 2), in the seasonally dry forest, there was a strongly significant interaction between species distribution and drought (Table 2); widespread species showed a very marginal, though non-significant negative response to herbivory, whereas the aseasonal species showed a very strong, significant negative response. This suggests that the impact of herbivory may be exacerbated by drought stress in aseasonal species. This result was only evident during one of the two census periods that included drought, but that period was a particularly strong drought event with near-drought conditions, initiating early and 1 month with virtually no rainfall (see methods). This preliminary finding fits with the idea that stressed plants or those experiencing resource limitations will have a reduced capacity to compensate for or tolerate herbivory (Coley et al. 1985; Louda et al. 1990; Gianoli et al. 2009). There are several reports on the interactive effects of herbivory and drought on plant fitness with equivocal findings (Hawkes and Sullivan 2001; Wise and Abrahamson 2007; Gonzales et al. 2008; Gianoli et al. 2009). A recent experimental study by Gianoli et al. (2009) demonstrated that leaf damage reduced the capacity of seedlings to respond plastically to reduced soil moisture. Specifically, in water-stressed seedlings, herbivory resulted in a lesser increase in water-use efficiency and root-shoot ratio and impacted changes in water potential, reducing drought tolerance, which resulted in lower survivorship of plants experiencing both drought and leaf damage (Gianoli et al. 2009). We do not have measures of the physiological status of seedlings from this field experiment, so we can only speculate on the mechanism underlying this interaction between drought and herbivory; further investigation of this finding would be warranted.

### Implications for biodiversity under predicted climate change

Tropical forests are one of the richest species assemblages on earth due in part to the warm, wet, and very stable climatic characteristic of these regions. Climate models are consistently indicating that anthropogenically driven climate change will result in more frequent and severe water deficits in these regions during this century (e.g., Williams et al. 2007), which has important implications for these hyperdiverse assemblages adapted to everwet conditions. Clearly, understanding the responses of the species that comprise this diversity is critical. In this manuscript, we provide evidence of two mechanisms that may contribute importantly to the fates of forest tree species along the floristically rich Thai-Malay Peninsula. First, widespread species that experience a broader range of climatic conditions show significantly greater survivorship in the face of drought, attributable to morphological and physiological traits conducive to drought tolerance. There is increasing evidence pan-tropically of trait-based performance and distribution differences in trees with respect to drought (Engelbrecht et al. 2007; Poorter and Markesteijn 2008; Brenes-Arguedas et al. 2009; Markesteijn and Poorter 2009). This should clearly be a critical focus, as our understanding of these differences will form an important basis in predictions of tropical forest responses to climate change. A second, novel mechanism that requires further investigation is that of drought–herbivore interactions. We provide evidence that drought may exacerbate the negative impact of herbivory on seedling survival, but that this impact is targeting species “specialized” to aseasonal forests (aseasonal species), suggestive of a potentially important role of insect herbivores in responses of aseasonal forests to predicted climate change. At least 60% of tree species in the 50-ha forest dynamics plot at Pasoh have distributions restricted to aseasonal forests (Baltzer et al. 2007). The implication of the present findings is that if aseasonal forests experience either increased frequency or severity of drought, systematic shifts in species composition and corresponding reductions in richness may be expected. Widespread species or “climatic generalists” that currently co-occur with aseasonal forest species should be favored due to their enhanced capacity to tolerate drought and maintain this tolerance in the face of herbivory.

## Biosketches

Jennifer Baltzer is a Canada Research Chair in Forests and Global Change in Wilfrid Laurier University's Biology Department. Her primary interests are the responses of trees to environmental stresses and how this helps to shape species distributions at local and regional scales.

Stuart Davies is the Director of the Center for Tropical Science – Smithsonian Global Earth Observatory program based out of Washington, DC. He is interested in the ecology and evolution of tropical rain forest trees, long-term dynamics of forest ecosystems, and tropical plant systematics.

## Supporting Information

**Table S1**. List of study species, family, and distributions (Dist; N = Northern, S = southern, W = widespread) in relation to the Kangar-Pattani Line (Fig. 1). Location indicates the seed collection site (P, Pasoh; KC, Khao Chong); number of seed sources is indicated in parentheses. In the Dipeterocarpaceae, mast fruiting often did not allow accurate counts of the number of contributing trees; the numbers thus represent number of seed collection locations.

**Figure S1.** Distribution-based differences in height relative growth rates (RGR_h_) between congeneric species pairs. Aseasonal species have distributions restricted to aseasonal forests (Pasoh), whereas widespread species have distributions including seasonally dry forests (Khao Chong). Due to a significant forest × distribution interaction, data were split by forest and distribution-based differences were tested using randomized block ANOVA (random blocking term: genus; fixed treatment term: distribution). Species codes are as follows: APOR, *Aporosa*; NEOS, *Neoscortechinia*; PALA, *Palaquium*; PARA, *Parashorea*; SHOR, *Shorea*. Species details are in Table S1.

**Figure S2.** Distribution-based differences in seedling survivorship between congeneric species pairs. Species distributional groupings, analytical description, and species codes follow Fig. S1. Species details can be found in Table S1.

**Figure S3.** Distribution-based differences in leaf damage incurred over the course of the experiment between congeneric species pairs. Species distributional groupings, analytical description, and species codes follow Fig. S1. Species details can be found in Table S1.

**Figure S4**. Root, stem, and leaf mass ratios (RMR, LMR, and SMR, respectively) as a function of species distribution and forest type. Differences were analyzed using linear mixed models with the fixed factor being a single combined category for forest and distribution; random factors included species and plot. Lower case letters indicate results from a Tukey test (*P* < 0.05). Aseasonal species have distributions restricted to aseasonal forests (Pasoh), whereas widespread species have distributions including seasonally dry forests (Khao Chong). Values represent averages across species ±1 SE over 19 months.

**Figure S5.** Mean seedling survivorship (±SE) by census and distributional category (aseasonal species means, open bars; widespread species means, black bars) with seasonal drought period in Khao Chong overlaid (gray panels). Species distributional groupings follow Fig. S1. We used linear mixed models to examine differences in mean plot-based survivorship among census intervals. Survival was calculated as the proportion of seedlings remaining from the previous census. Due to significant interaction terms, data were split by forest and run separately. The design included two fixed effects: species distribution and census interval, and two random factors: species and plot. Survivorship data were square-root arc-sin transformed for analyses. In Pasoh, seedling survival varied among censuses (*P* < 0.0001) and aseasonal species had consistently greater survival than widespread species (*P* = 0.0048) regardless of census (Census × Dist *P* = 0.1775). In Khao Chong, survivorship was likewise variable among censuses (*P* < 0.0001), but a significant census × distribution interaction was detected (*P* = 0.0016) with widespread species having greater survivorship during and immediately following drought, but not in other census periods.
