# Supplementary material for: Rainfall seasonality and pest pressure as determinants of tropical tree species' distributions
Source: Ecol Evol. 2012 Sep 27;2(11):2682–94. doi: 10.1002/ece3.383 (PMC3501622; doi:10.1002/ece3.383)
Supplement: Supplementary file 1 [file ece30002-2682-SD6.doc]

**Table S1**. List of study species, family and distributions (Dist; N = Northern, S = southern, W = widespread) in relation to the Kangar-Pattani Line (Fig. 1). Location indicates the seed collection site (P, Pasoh; KC, Khao Chong); number of seed sources is indicated in parentheses. In the Dipeterocarpaceae, mast fruiting often did not allow accurate counts of the number of contributing trees; the numbers thus represent number of seed collection locations.

| **Family** | **Species** | **Dist** | **Location** |
| --- | --- | --- | --- |
| Dipterocarpaceae | *Parashorea stellata* Kurz. | N | KC(5) |
|  | *Parashorea densiflora* (Y.Sl.) ex Sym. | S | P(1) |
|  | *Shorea guiso* (Blanco) Blume | W | P(2) |
|  | *Shorea macroptera* Dyer | S | P(>3) |
|  | *Shorea parvifolia* Dyer | S | P(>3) |
|  | *Vatica odorata* (Griff.) Symington | W | KC(2) |
| Euphorbiaceae | *Mallotus penangensis* Müll. Arg. | S | P(3) |
|  | *Neoscortechinia kingii* Hk.f. | S | P(2) |
|  | *Neoscortechnia philippinensis* (Merr.) Welzen | W | KC(2) |
| Fagaceae | *Quercus semiserrata* Roxb. | N | KC(2) |
| Phyllanthaceae | *Aporosa microstachya* Hook. F | W | P(5) |
|  | *Aporosa symplocoides* (hook f.) paqx | S | P(3) |
| Sapotaceae | *Palaquium maingayi* K&G | S | P(3) |
|  | *Palaquium sumatrana* Burck. | W | KC(2) |
|  | *Payena lucida* (Don) DC | W | P(>3) |
| Violiaceae | *Rinorea anguifera* (Lour.) OK | W | P(8) |
