# Supplementary material for: Rainfall seasonality and pest pressure as determinants of tropical tree species' distributions
Source: Ecol Evol. 2012 Sep 27;2(11):2682–94. doi: 10.1002/ece3.383 (PMC3501622; doi:10.1002/ece3.383)
Supplement: Supplementary file 2 [file ece30002-2682-SD1.docx]

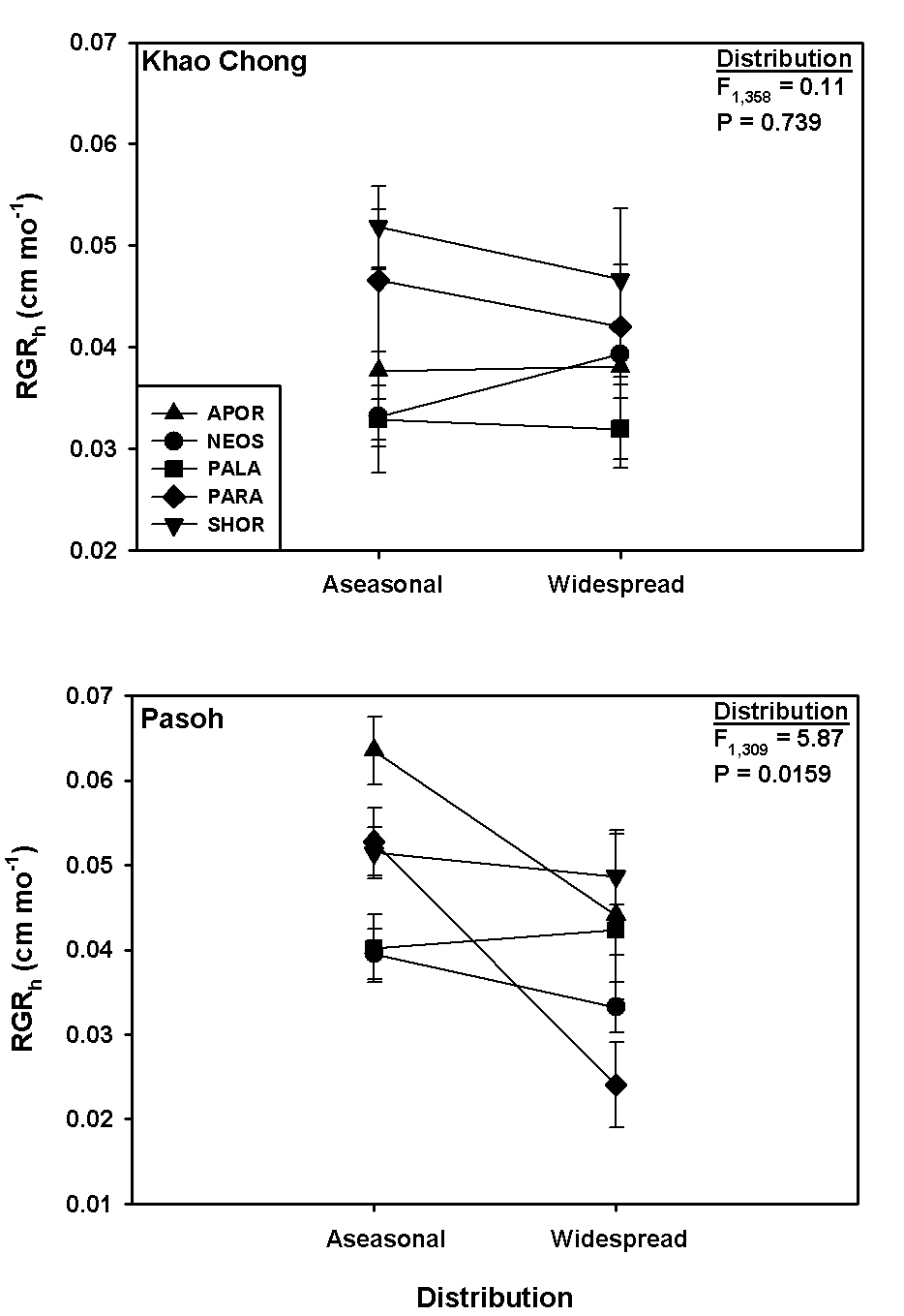


**Figure S1.** Distribution-based differences in height relative growth rates (RGR_h_) between congeneric species pairs. Aseasonal species have distributions restricted to aseasonal forests (Pasoh), while widespread species have distributions including seasonally dry forests (Khao Chong). Due to a significant forest*distribution interaction, data were split by forest and distribution-based differences were tested using randomized block ANOVA (random blocking term: genus; fixed treatment term: distribution). Species codes are as follows: APOR, *Aporosa*; NEOS, *Neoscortechinia*; PALA, *Palaquium*; PARA, *Parashorea*; SHOR, *Shorea*. Species details are in Table S1.


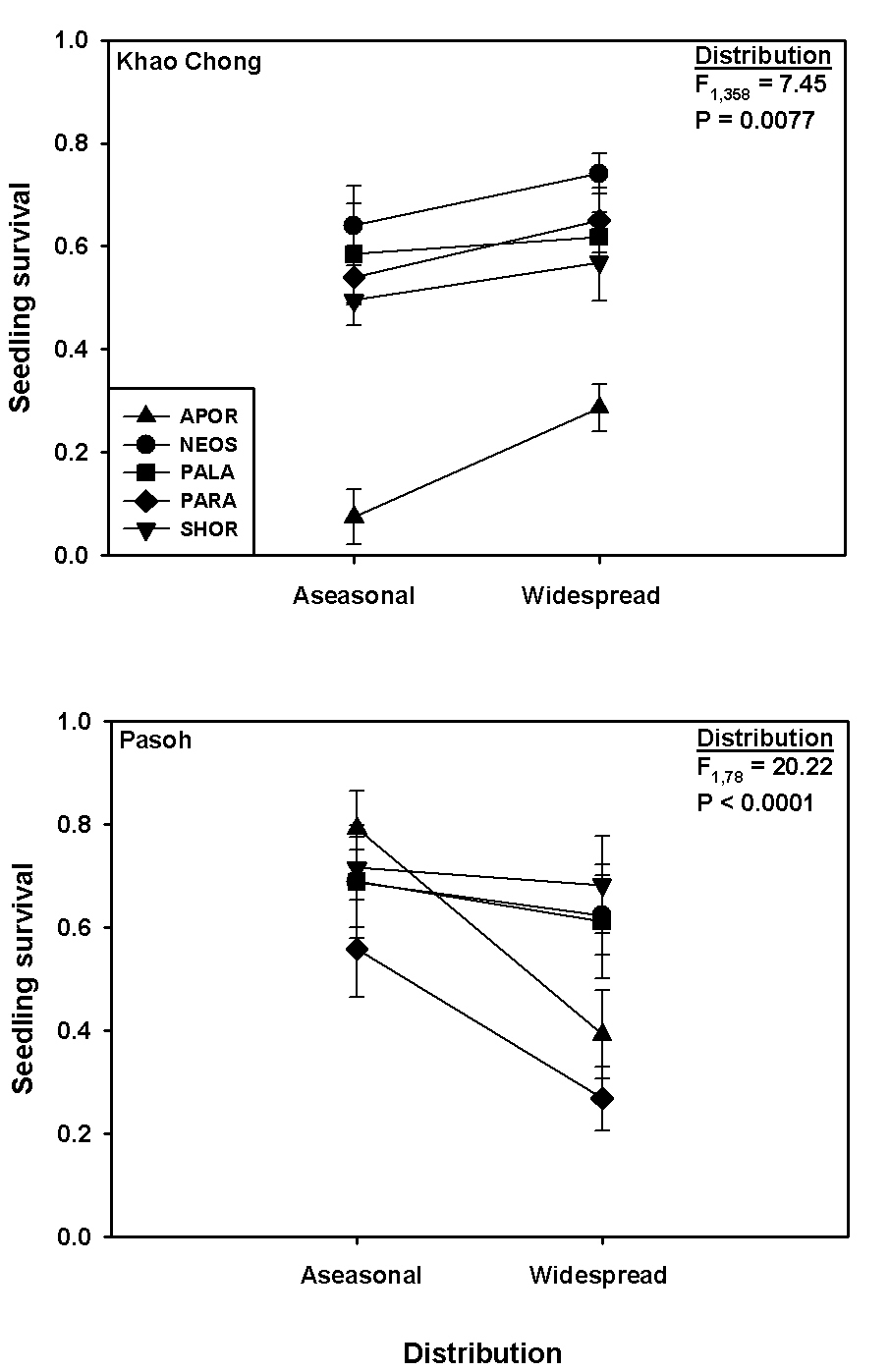


**Figure S2.** Distribution-based differences in seedling survivorship between congeneric species pairs. Species distributional groupings, analytical description and species codes follow Fig. S1. Species details can be found in Table S1.


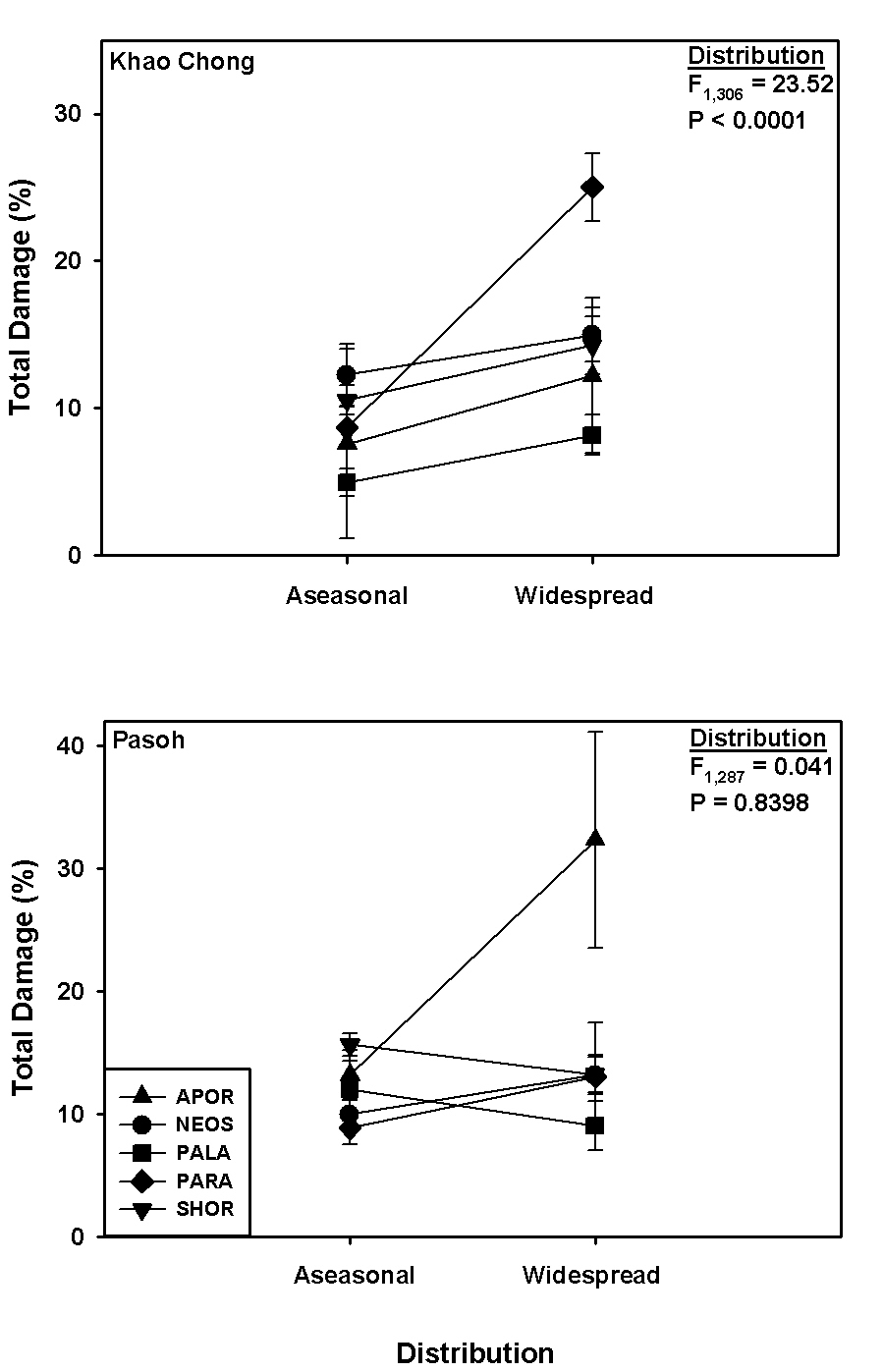


**Figure S3.** Distribution-based differences in leaf damage incurred over the course of the experiment between congeneric species pairs. Species distributional groupings, analytical description and species codes follow Fig. S1. Species details can be found in Table S1.


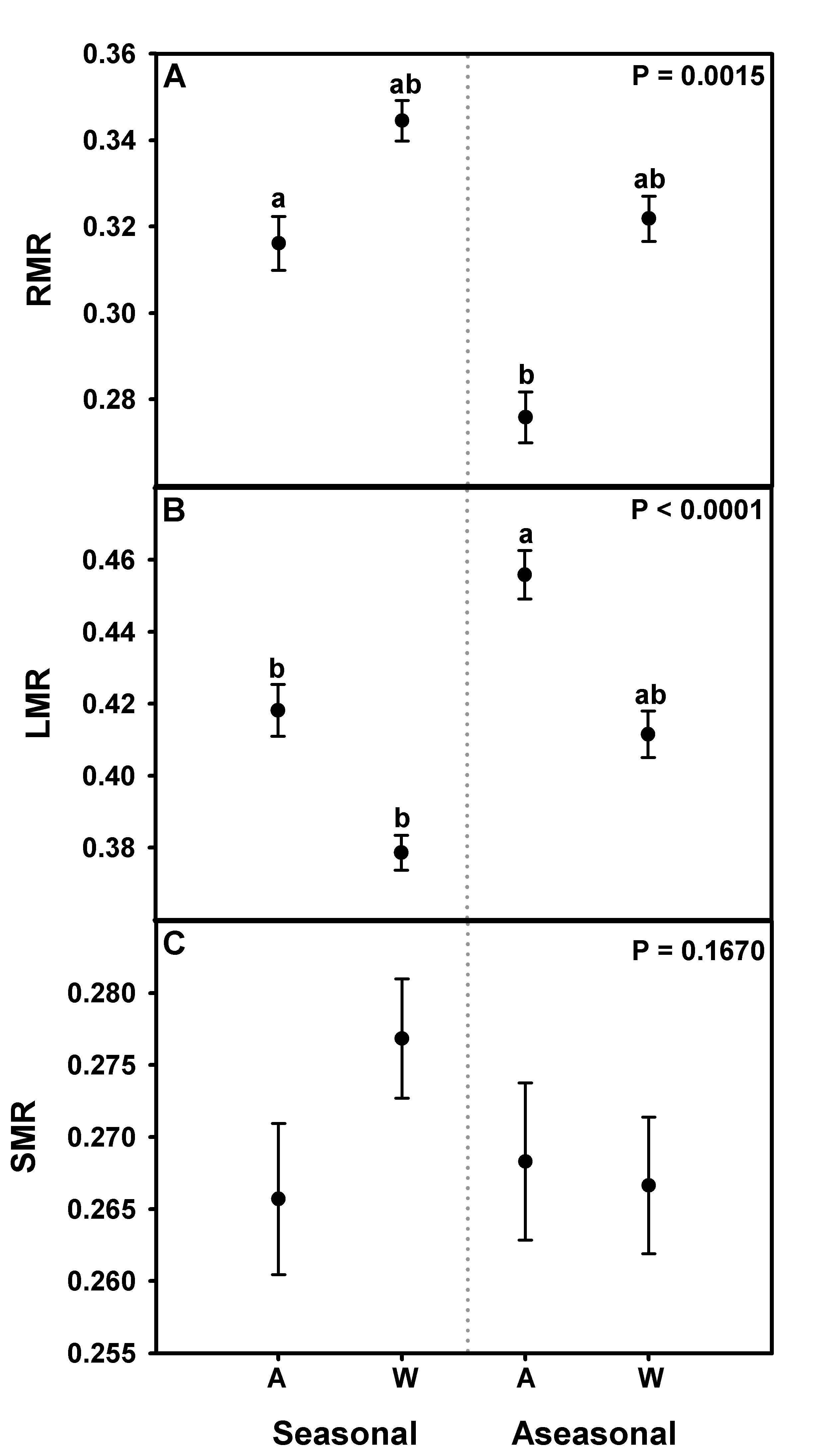


**Figure S4**. Root, stem and leaf mass ratios (RMR, LMR, and SMR, respectively) as a function of species distribution and orest type. Differences were analyzed using linear mixed models with the fixed factor being a single combined category for forest and distribution; random factors included species and plot. Lower case letters indicate results from a Tukey test (P < 0.05). Aseasonal species have distributions restricted to aseasonal forests (Pasoh), while widespread species have distributions including seasonally dry forests (Khao Chong). Values represent averages across species ± 1 SE over 19 months.


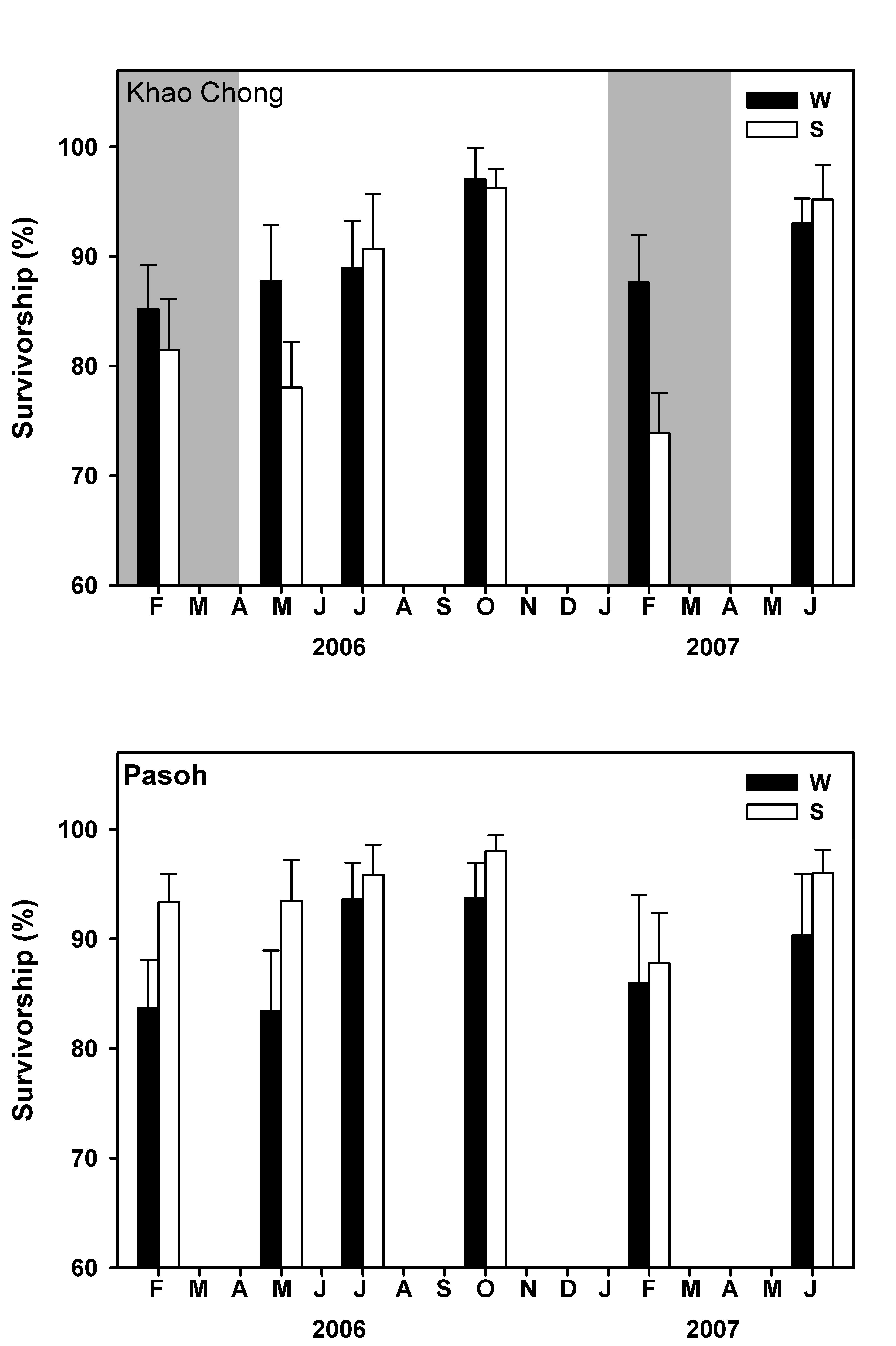


**Figure S5.** Mean seedling survivorship (±SE) by census and distributional category (aseasonal species means, open bars; widespread species means, black bars) with seasonal drought period in Khao Chong overlaid (grey panels). Species distributional groupings follow Fig. S1. We used linear mixed models to examine differences in mean plot-based survivorship among census intervals. Survival was calculated as the proportion of seedlings remaining from the previous census. Due to significant interaction terms, data were split by forest and run separately. The design included two fixed effects: species distribution and census interval, and two random factors: species and plot. Survivorship data were square-root arc-sin transformed for analyses. In Pasoh, seedling survival varied among censuses (P < 0.0001) and aseasonal species had consistently greater survival than widespread species (P = 0.0048) regardless of census (Census*Dist P = 0.1775). In Khao Chong, survivorship was likewise variable among censuses (P < 0.0001) but a significant census*distribution interaction was detected (P = 0.0016) with widespread species having greater survivorship during and immediately following drought but not in other census periods.
